# Cytogenetic and symbiont analysis of five members of the *B. dorsalis* complex (Diptera, Tephritidae): no evidence of chromosomal or symbiont-based speciation events

**DOI:** 10.3897/zookeys.540.9857

**Published:** 2015-11-26

**Authors:** Antonios A. Augustinos, Elena Drosopoulou, Aggeliki Gariou-Papalexiou, Elias D. Asimakis, Carlos Cáceres, George Tsiamis, Kostas Bourtzis, Penelope Mavragani-Tsipidou, Antigone Zacharopoulou

**Affiliations:** 1 Department of Biology, University of Patras, Patras, Greece; 2 Insect Pest Control Laboratory, Joint FAO/IAEA Programme of Nuclear Techniques in Food and Agriculture, Seibersdorf, Vienna, Austria; 3 Department of Environmental and Natural Resources Management, University of Patras, Agrinio, Greece; 4 Department of Genetics, Development and Molecular Biology, School of Biology, Faculty of Sciences, Aristotle University of Thessaloniki, Thessaloniki, Greece

**Keywords:** Tephritidae, *Wolbachia*, inversions, polytene chromosomes

## Abstract

The *Bactrocera dorsalis* species complex, currently comprising about 90 entities has received much attention. During the last decades, considerable effort has been devoted to delimiting the species of the complex. This information is of great importance for agriculture and world trade, since the complex harbours several pest species of major economic importance and other species that could evolve into global threats. Speciation in Diptera is usually accompanied by chromosomal rearrangements, particularly inversions that are assumed to reduce/eliminate gene flow. Other candidates currently receiving much attention regarding their possible involvement in speciation are reproductive symbionts, such as *Wolbachia*, *Spiroplasma*, *Arsenophonus*, *Rickettsia* and *Cardinium*. Such symbionts tend to spread quickly through natural populations and can cause a variety of phenotypes that promote pre-mating and/or post-mating isolation and, in addition, can affect the biology, physiology, ecology and evolution of their insect hosts in various ways. Considering all these aspects, we present: (a) a summary of the recently gained knowledge on the cytogenetics of five members of the *Bactrocera dorsalis* complex, namely *Bactrocera dorsalis* *s.s.*, *Bactrocera invadens*, *Bactrocera philippinensis*, *Bactrocera papayae* and *Bactrocera carambolae*, supplemented by additional data from a *Bactrocera dorsalis* *s.s.* colony from China, as well as by a cytogenetic comparison between the *dorsalis* complex and the genetically close species, *Bactrocera tryoni*, and, (b) a reproductive symbiont screening of 18 different colonized populations of these five taxa. Our analysis did not reveal any chromosomal rearrangements that could differentiate among them. Moreover, screening for reproductive symbionts was negative for all colonies derived from different geographic origins and/or hosts. There are many different factors that can lead to speciation, and our data do not support chromosomal and/or symbiotic-based speciation phenomena in the taxa under study.

## Introduction

The *Bactrocera dorsalis* species complex currently consists of approximately 90 entities, whose limits are not fully resolved (Drew and Hancock 1994, Drew and Romig 2013, Krosch et al. 2013, Boykin et al. 2014, Schutze et al. 2015). However, species delimitation is of paramount importance when dealing with economic important species, since it can influence world trade through implementation of quarantine policies and/or facilitate the application of species specific, environmental friendly control methods, such as the Sterile Insect Technique (SIT). Driven by these considerations, much effort has been invested in the last decades to clarify the species status within the complex. Among the most recent advances in this area, Drew and Romig (2013) synonymised *Bactrocera papayae* and *Bactrocera philippinensis* under *Bactrocera papayae*, while Schutze and colleagues (Scutze et al. 2015) have proposed the further synonymization of these two taxa and *Bactrocera invadens* with *Bactrocera dorsalis* *s.s.*, under *Bactrocera dorsalis* *s.s.*

Recent studies have shown that efforts to resolve complex species status require multidisciplinary approaches (De Queiroz 2007, Schlick-Steiner et al. 2010), well-characterized material and extended sampling (Schutze et al. 2012, Krosch et al. 2013, Boykin et al. 2014). Such approaches have been also followed in other Tephritidae genera where species delimitation of species complexes is also an important concern, such as in *Anastrepha* (Selivon et al. 2005, Vera et al. 2006, Cáceres et al. 2009). This is due to the fact that speciation can be driven by a variety of forces, resulting in different speciation paths. The data basis can be complicated when speciation is ongoing (incipient). Therefore, in collaboration and through independent analysis, different research groups around the world, through the Coordinated Research Program: ‘Resolution of Cryptic Species Complexes of Tephritid Pests to Enhance SIT Application and Facilitate International Trade’ have accumulated a multitude of data that have contributed to the better understanding of the Tephritidae species complexes. One of the main targets was the resolution among five economic important taxa with unclear limits within the *Bactrocera dorsalis* complex. These were *Bactrocera dorsalis* *s.s.*, *Bactrocera papayae*, *Bactrocera philippinensis*, *Bactrocera invadens* and *Bactrocera carambolae*.

A key pathway of speciation in Diptera is through chromosomal rearrangements (CRs), mainly inversions. More than fifty years of research on polytene chromosomes of *Drosophila* and mosquito species have shown that speciation is almost universally accompanied with inversions (Sturtevant and Dobzhansky 1936, Ashburner et al. 1982, Krimbas and Powell 1992, Noor et al. 2001, Rieseberg 2001, Kirkpatrick and Barton 2006, Bhutkar et al. 2008, Kulathinal et al. 2009, Stevison et al. 2011, Lee et al. 2013). The recent advances in whole genome sequencing and the availability of a number of genomes of *Drosophila* and mosquito species have verified the nuclear DNA rearrangements described in earlier cytogenetic studies (Kirkpatrick and Barton 2006, Ranz et al. 2007, Bhutkar et al. 2008, Schaeffer et al. 2008, Kulathinal et al. 2009, McGaugh and Noor 2012, Lee et al. 2013). Different models have been proposed to explain how CRs enhance speciation, recently focusing mainly on the restriction of recombination within and near inverted regions as the causal factor of restriction in gene flow (Noor et al. 2001, Rieseberg 2001, Kirkpatrick and Barton 2006, Faria and Navarro 2010).

However, sequencing of entire genomes cannot yet be easily applied to species with bigger genomes and a high proportion of repetitive DNA sequences. Shotgun sequencing approaches are relatively quick and cheap, but cannot provide insight into higher chromosomal organization of species lacking of a complete sequenced reference genome, at least up to now. Regarding the *Bactrocera dorsalis* complex, the draft genome of *Bactrocera dorsalis* *s.s.* currently consists of more than 86,000 contigs (http://www.ncbi.nlm.nih.gov/assembly/GCF_000789215.1). Even though the construction of several genome databases of Tephritidae species is ongoing, this methodology is so far (a) too slow and expensive to screen a large number of different populations and (b) it is not guaranteed to reveal structural chromosomal changes between species, unless coupled with molecular and genetic approaches, such as Sanger sequencing, cloning and *in situ* hybridization. Direct observation and comparison of chromosomes is still a very powerful approach to shed light on the higher organization and structure of chromosomes. Although mitotic chromosomes can also provide some information, polytene chromosomes are an excellent tool for resolution of CRs.

In Tephritids, there is a number of studies presenting and discussing mitotic karyotypes, especially for *Bactrocera* (Hunwattanakul and Baimai 1994, Baimai et al. 1995, 1999, 2000, Baimai 1998, 2000), *Anastrepha* (Cevallos and Nation 2004, Selivon et al. 2005, Goday et al. 2006, Selivon et al. 2007) and *Rhagoletis* species (Bush and Boller 1977). However, useful polytene chromosome maps, so far available for five genera, represent only 11 species: one of *Anastrepha* (*Anastrepha ludens*) (Garcia-Martinez et al. 2009), one of *Ceratitis* (*Ceratitis capitata*) (Zacharopoulou 1990), one of *Dacus* (*Dacus ciliatus*) (Drosopoulou et al. 2011b) and three of *Rhagoletis*, namely *Rhagoletis cerasi* (Kounatidis et al. 2008), *Rhagoletis cingulata* (Drosopoulou et al. 2011a) and *Rhagoletis completa* (Drosopoulou et al. 2010). The genus *Bactrocera* can be regarded as the best studied so far, including four species of three different subgenera. These are *Bactrocera oleae* (subgenus *Daculus*) (Mavragani-Tsipidou et al. 1992), *Bactrocera cucurbitae* (subgenus *Zeugodacus*) (Zacharopoulou et al. 2011b) and *Bactrocera dorsalis* *s.s.* (Zacharopoulou et al. 2011a) plus *Bactrocera tryoni* (subgenus *Bactrocera*) (Zhao et al. 1998).

Cytogenetic studies have been used to distinguish between different members of the *Bactrocera dorsalis* complex in the past, based on mitotic chromosomes. Hunwattanakul and Baimai (1994) presented the typical karyotype of *Bactrocera dorsalis*, which is being referred to as form A. The mitotic karyotype of the complex is 2n = 12, consisting of five pairs of autosomes and a heterogametic XX/XY sex chromosome pair. In the following years, Baimai and colleagues presented numerous species within the complex with distinct mitotic karyotypes (Baimai et al. 1995, 2000, Baimai 1998). Although these studies are of great importance and reveal the power of cytogenetics for the resolution of species limits within species complexes, they suffered from limitations that could not be addressed or even predicted in the previous years. These include (a) the ongoing debate on species limits and taxonomy of the complex, (b) utilization of material from the field that cannot be evaluated with other approaches, since it was not colonized and, (c) lack of robust diagnostic tools within this complex. All these indicate that older taxonomic conclusions should be used with care and seen in the light of recent advances in the field.

To overcome such constraints, recent cytogenetic studies have used laboratory colonies from the Joint FAO/IAEA Insect Pest Control Laboratory (IPCL). These colonies are also material in a variety of research programs, are always available for further analyses and their status is routinely verified by expert taxonomists. Zacharopoulou and colleagues analysed colonized material of *Bactrocera dorsalis* *s.s.*, derived from Thailand and from a Genetic Sexing Strain (GSS) constructed in Hawaii (Zacharopoulou et al. 2011a). In this study, the form A mitotic karyotype was verified for *Bactrocera dorsalis* *s.s.*, and polytene chromosome map for this species was constructed, which includes 10 polytene arms. These arms correspond to the autosomes, which is consistent with the already described non-polytenization of the sex chromosomes in Tephritidae (Zacharopoulou 1990, Mavragani-Tsipidou et al. 1992, Zhao et al. 1998, Garcia-Martinez et al. 2009, Drosopoulou et al. 2010, Zacharopoulou et al. 2011a, Zacharopoulou et al. 2011b, Drosopoulou et al. 2011a, Drosopoulou et al. 2011b, Drosopoulou et al. 2012). Recently, a more extended cytogenetic analysis was performed (Augustinos et al. 2014b), shedding more light on the resolution of the species limits of the five taxa described before. Six laboratory colonies, representing *Bactrocera dorsalis* *s.s.* (two colonies), *Bactrocera papayae*, *Bactrocera philippinensis*, *Bactrocera invadens* and *Bactrocera carambolae*, were examined (Table 1) and all exhibited the form A mitotic karyotype. This was quite a surprise, since it is not in agreement with previous studies, where a distinct karyotype with a quite large X chromosome, carrying an ‘elongated’ arm with a secondary constriction, was described for *Bactrocera carambolae* from Thailand (Baimai et al. 1999). In addition, polytene chromosomes did not reveal any fixed CRs among these five taxa that could be used as diagnostic markers (Augustinos et al. 2014b).

**Table 1.** Material used in the present study.

No	Species	Origin	Reproductive symbiont screening*		Cytogenetically analyzed
			M	F	
1	*Bactrocera dorsalis*	Saraburi, Thailand	10	10	Zacharopoulou et al. 2011a Augustinos et al. 2014b
2	*Bactrocera dorsalis*	Nakhon Sri Thammarat, Thailand	10	10	Augustinos et al. 2014b
3	*Bactrocera dorsalis G17*	Bangkok, Thailand	10	10	
4	*Bactrocera dorsalis GSS*	Hawaii	10	10	Zacharopoulou et al. 2011a; Zacharopoulou and Franz 2013
5	*Bactrocera dorsalis* (*White body*)	OAP, Bangkok, Thailand	10	10	
6	*Bactrocera dorsalis*	Yunnan, China	10	10	
7	*Bactrocera dorsalis*	Fujian, china	10	10	
8	*Bactrocera dorsalis*	Pakistan	10	10	
9	*Bactrocera dorsalis*	Myanmar	10	10	
10	*Bactrocera dorsalis*	India	10	10	
11	*Bactrocera dorsalis*	Wuhan, China (colony 1)	10	10	Present study
12	*Bactrocera dorsalis*	Wuhan, China (colony 2)	10	10	
13	*Bactrocera carambolae*	Paramaribo, Suriname	10	10	Augustinos et al. 2014b
14	*Bactrocera carambolae*	Serdang, Malaysia	10	10	
15	*Bactrocera philippinensis*	Guimaras Island, Philippines	10	10	
16	*Bactrocera philippinensis*	Philippines	10	10	Augustinos et al. 2014b
17	*Bactrocera papayae*	Serdang, Malaysia	10	10	Augustinos et al. 2014b
18	*Bactrocera invadens*	Kenya	10	10	Augustinos et al. 2014b
19	*Bactrocera tryoni*	Australia	10	10	Present study

*Twenty flies were screened for the presence of the five reproductive symbionts listed in Table 2. None was positive for none of the symbionts.

A second factor that should not be overlooked in studies addressing speciation phenomena is the presence of specific symbiotic bacteria, especially those referred to as ‘reproductive parasites’. These are symbiotic bacteria mainly found in reproductive tissues and are best known to interfere with host reproduction, inducing a variety of phenotypes such as male killing, parthenogenesis, feminization and Cytoplasmic Incompatibility (CI). Among them, *Cardinium*, *Arsenophonus*, *Spiroplasma*, *Rickettsia* and *Wolbachia* are commonly found in different arthropods (Bourtzis and Miller 2003, 2006, 2009, Perlman et al. 2006, Duron et al. 2008a, Werren et al. 2008, Saridaki and Bourtzis 2010, Zchori-Fein and Bourtzis 2011).

*Wolbachia* is probably the most ubiquitous bacterial symbiont in insects (Hilgenboecker et al. 2008, Zug and Hammerstein 2012) and is regarded as a putative ‘speciation agent’, since it can restrict gene flow through (CI) and lead to the selection and fixation of specific genotypes in a population. *Wolbachia*-induced CIs can co-exist with local selection on alleles involved in incompatibilities and, therefore, increase the migration rates that genetic variability can experience without getting lost. The combined act of the two aforementioned forces of incompatibility can lead to maintenance of the divergence among populations and enhance speciation (Flor et al. 2007, Telschow et al. 2007, 2014). Besides theoretical and model predictions, the implication of *Wolbachia* in pre- and / or post-mating isolation phenomena has been experimentally supported in different insect systems including the parasitic wasps of the genus *Nasonia* (Bordenstein et al. 2001, Bordenstein and Werren 2007) and *Drosophila* (Jaenike et al. 2006, Koukou et al. 2006, Miller et al. 2010).

In tephritids, most studies have so far focused on the detection and characterization of *Wolbachia* infections. Although screening is far from complete, well-established infections have been found in some species. The best characterized species is *Rhagoletis cerasi*, since all natural populations studied so far are 100% infected, usually with multiple-strain infections (Riegler and Stauffer 2002, Kounatidis et al. 2008, Arthofer et al. 2009, Augustinos et al. 2014a, Karimi and Darsouei 2014). More importantly, it is a well-documented example of the implication of *Wolbachia* in restriction in gene flow and enhancement of incompatibility between natural populations of the species (Riegler and Stauffer 2002). Other *Rhagoletis* species that seem to have persistent and multiple strain infections (although less populations are studied) are *Rhagoletis pomonella* (Schuler et al. 2011) and *Rhagoletis cingulata* (Drosopoulou et al. 2011a, Schuler et al. 2013), along with some *Rhagoletis* species of Japan (Coats et al. 2013). Outside *Rhagoletis*, the only species demonstrating persistent *Wolbachia* infections is *Anastrepha fraterculus*, (Selivon et al. 2002, Cáceres et al. 2009, Coscrato et al. 2009, Marcon et al. 2011, Martinez et al. 2012). All other tephritid species are so far considered as *Wolbachia*-free or only exhibiting low prevalence infections. Among them, *Ceratitis capitata* is also considered as *Wolbachia*-free (Bourtzis et al. 1994, Zabalou et al. 2004); however, there are two reports from a research group in Latin America discussing the presence of *Wolbachia* in local populations of the species (Rocha et al. 2005, Coscrato et al. 2009). The recent study on the *Wolbachia* presence in Australian fruit flies (Morrow et al. 2015) has extended our knowledge on the *Wolbachia* status of Tephritidae in a relatively unexplored area. In accordance with previous studies, few species were found infected and only a relatively small (although varying) percentage of individuals. However, this study demonstrated the presence of different *Wolbachia* strains, shared among natural populations of different species, raising the possibility of recent horizontal transmission events through shared parasitoids. Regarding the other four symbionts, there are up to now no reports of infected populations, at least to our knowledge. Especially for the *Bactrocera dorsalis* complex, there are only three reports of *Wolbachia* infections in natural populations. In all these cases, infections were found at a very low prevalence in nature (Kittayapong et al. 2000, Jamnongluk et al. 2002, Sun et al. 2007).

The purpose of this study was to (a) summarize gained knowledge and (b) provide new evidence regarding the cytogenetic and symbiotic status of the *Bactrocera dorsalis* complex, with the aim to identify factors possibly involved in speciation. Focus has been given on five taxa of economic importance and unclear species limits, namely *Bactrocera dorsalis* *s.s.*, *Bactrocera papayae*, *Bactrocera philippinensis*, *Bactrocera invadens* and *Bactrocera carambolae*. Only material colonized at the Joint FAO/IAEA IPCL was analysed, that was also used in other Joint FAO/IAEA IPCL research programs (Wee et al. 2002, Krosch et al. 2013, Boykin et al. 2014, Schutze et al. 2013, Tan et al. 2013, Bo et al. 2014). More specifically, our cytogenetic analysis was extended to (a) a *Bactrocera dorsalis* *s.s.* population derived from China, a cytogenetically unexplored area of great interest for the complex, (b) a new Australian colony of *Bactrocera tryoni*, a species that is genetically discrete though not phylogenetically distant from the *Bactrocera dorsalis* complex and (c) F1 bidirectional hybrids of *Bactrocera dorsalis* *s.s.* and *Bactrocera tryoni*. In addition, an extensive PCR screening was performed aiming at the detection of the five aforementioned reproductive symbionts in 18 different colonies available for the *dorsalis* complex and the colony representing *Bactrocera tryoni* (Table 1).

## Methods

### Material used

Nineteen colonies currently kept at the Joint FAO/IAEA IPCL were screened for the presence of different reproductive symbionts (Table 1). Eighteen of them represent the five members of the complex under discussion (*Bactrocera dorsalis* *s.s.*, *Bactrocera papayae*, *Bactrocera philippinensis*, *Bactrocera invadens* and *Bactrocera carambolae*), while one colony represents *Bactrocera tryoni* from Australia that was included as a closely related outgroup. Two colonies were cytogenetically analysed (*Bactrocera dorsalis* *s.s.* from China-Wuhan and *Bactrocera tryoni* from Australia) and were added to the seven colonies previously analysed (see Table 1 and references therein). The F_1_ bidirectional hybrids of *Bactrocera dorsalis* *s.s.* × *Bactrocera tryoni* were also analysed.

### Mitotic chromosome preparations

Chromosome preparations were made as described in Zacharopoulou (1990) and Mavragani-Tsipidou et al. (2014). Brain tissue was dissected in 0.7% NaCl, transferred to 1% sodium citrate on a well slide for at least 15 min and fixed in fresh fixation solution (methanol/acetic acid 3:1) for 3 min. Fixation solution was removed and a drop of acetic acid (60%) was added. Tissue was dispersed using a micropipette and the cell suspension was dried on a clean slide placed on a hotplate (40–45 °C). Chromosomes were stained with Giemsa (5% Giemsa in 10 mM phosphate buffer, pH 6.8). Chromosome slides were analysed at 100× magnification, using a phase contrast microscope (Leica DMR), and photographs were taken using a CCD camera (ProgRes CFcool; Jenoptik Jena Optical Systems, Jena, Germany). At least 15 good quality preparations per sample and at least 10 well-spread nuclei per preparation were analysed.

### Polytene chromosome preparations

Polytene chromosome preparations were made from 3rd instar larvae, as described in Zacharopoulou (1990), Mavragani-Tsipidou et al. (2014). Larvae were dissected in acetic acid (45%), and salivary glands were transferred to HCl (3 N) for 1 min, fixed in 3:2:1 fixation solution (3 parts acetic acid: 2 parts water: 1 part lactic acid) for ~5 min (until transparent) and stained in 2% lacto-aceto-orcein for 5–7 min. Glands were washed with 3:2:1 solution to remove excess stain and squashed. Chromosome slides were analysed at 100× magnification using a phase contrast microscope (Leica DMR) and photographs were taken using a CD camera (ProgRes CFcool; Jenoptik Jena Optical Systems, Jena, Germany). At least 15 good quality preparations per sample and at least 10 well spread nuclei per preparation were analysed.

### DNA extraction and PCR screening for reproductive symbionts

DNA was extracted from single flies, using the CTAB protocol (Doyle and Doyle 1990). To verify DNA quality, PCRs were performed for randomly selected samples with the universal primer pair 12SCFR/12SCRR that amplifies 420 bp of the insect mitochondrial 12S rRNA gene (Hanner and Fugate 1997). In total, 380 samples were screened for the presence of *Wolbachia*, *Spiroplasma*, *Arsenophonus*, *Rickettsia* and *Cardinium*. Screening was performed using bacterial species-specific 16S *rRNA* gene-based PCR. Depending on the set of primers used, the amplified DNA fragment varied in size from 200 bp to 611 bp. The amplification was performed in 20 µl reactions, each containing 2 µl of 10× KAPA *Taq* Polymerase Buffer A (with 1.5 mM of MgCl_2_ at 1×), 0.1 µl of dNTPs (25 mM), 0.5 µl of the forward primer (25 µM), 0.5 µl of the reverse primer (25 µM), 0.1 µl of KAPA *Taq* DNA Polymerase (5 U/µl), 15.8 µl of sterile double distilled water and 1 µl of DNA. The PCR protocol included an initial 5 minute denaturation at 95 °C, followed by 35 cycles of 30 seconds at 95 °C, 30 seconds at the optimum melting temperature for each pair of specific primers, 1 minute at 72 °C and a final extension step of 10 minutes at 72 °C, with the exception of *Wolbachia*, where 30 cycles were used. The products were electrophoresed on a 1.5 % agarose gel in order to determine the presence and size of the fragments. Primer pairs and PCR conditions are summarized in Table 2.

**Table 2.** PCR screening for five reproductive symbionts.

Genus	Primer 5’-3’	Tm°C	Product Size	Reference
*Wolbachia*	wspecF YATACCTATTCGAAGGGATAG	55 °C	438 bp	Werren and Windsor 2000
	wspecR AGCTTCGAGTGAAACCAATTC			
*Spiroplasma*	63F_CG	60 °C	450 bp	Mateos et al. 2006 Fukatsu and Nikoh 2000
	GCCTAATACATGCAAGTCGAACGG			
	TKSSspR			
	TAGCCGTGGCTTTCTGGTAA			
*Arsenophonus*	ArsF	56 °C	611 bp	Duron et al. 2008b
	GGGTTGTAAAGTACTTTCAGTCGT			
	ArsR5			
	CCCTAAGGCACGYYTYTATCTCTAA			
*Rickettsia*	16SA1	55 °C	200 bp	Fukatsu and Nikoh 2000
	AGAGTTTGATCTGGCTCAG			
	Rick16SR			
	CATCCATCAGCGATAAATCTTTC			
*Cardinium*	CLO-f1	56 °C	466 bp	Gotoh et al. 2007
	GGAACCTTACCTGGGCTAGAATGTATT			
	CLO-r1			
	GCCACTGTCTTCAAGCTCTACCAAC			

## Results and discussion

As already stated in the Introduction, material colonized in IPCL was used in the present study. This is in the frame of utilizing multi-disciplinary approaches, using the same samples if possible, to contribute to the species resolution in the *dorsalis* complex (Schutze et al. 2015). For such approaches utilization of colonized, well-characterized material is essential. This is even more evident for cytogenetics, since live material is needed. On the other hand, results obtained from laboratory colonies must be verified in larger samples of different origin before elevating to species level. As it has been shown by different studies (Gilchrist et al. 2012; Parreño et al. 2014, Zygouridis et al. 2014), lab colonization is accompanied by an adaptation process including severe bottlenecks, hitch-hiking effects and extended inbreeding. This can affect the genetic structure of the populations and, possibly, their symbiotic communities. Therefore, results derived from colonized material should be ‘interpreted’ wisely and in combination with the analysis of natural collections.

### Mitotic karyotypes – agreements and inconsistencies with older studies

The *Bactrocera dorsalis* *s.s.* colony from China showed the *Bactrocera dorsalis* *s.s.* mitotic karyotype known as form A. This is the typical and probably ancestral karyotype of the *dorsalis* complex. The above, together with previous results, show that the Joint FAO/IAEA IPCL colonies, representing the five investigated taxa, possess the same mitotic karyotype (Zacharopoulou et al. 2011a, Zacharopoulou and Franz 2013, Augustinos et al. 2014b). Older studies (Baimai et al. 1999) describe a different karyotype for *Bactrocera carambolae* from Thailand. Although the *Bactrocera carambolae* colony analysed recently, available at the Joint FAO/IAEA IPCL (Augustinos et al. 2014b), was derived from a Suriname population, it is highly unlikely that the different origin is the explanation for this difference. Incorrect species identification due to the limitations discussed in the Introduction Section is the most probable explanation. This is further supported by the fact that an independent study on the mitotic karyotypes of *Bactrocera carambolae* from Malaysia also found the typical form A karyotype for this taxon (Yesmin and Clyde 2012).

The examination of new material representing *Bactrocera tryoni* from Australia was in accordance with the previously published mitotic karyotype for this species (Zhao et al. 1998). This karyotype has five pairs of autosomes and a heterogametic XX/XY sex chromosome pair. The three larger autosome pairs are metacentric to submetacentric, while the two shorter autosome pairs are submetacentric to acrocentric. Y is the smallest of the set, while X is large and probably larger than or comparable to the largest autosomes.

### Polytene chromosome comparisons and species resolution

**Figure 1.**
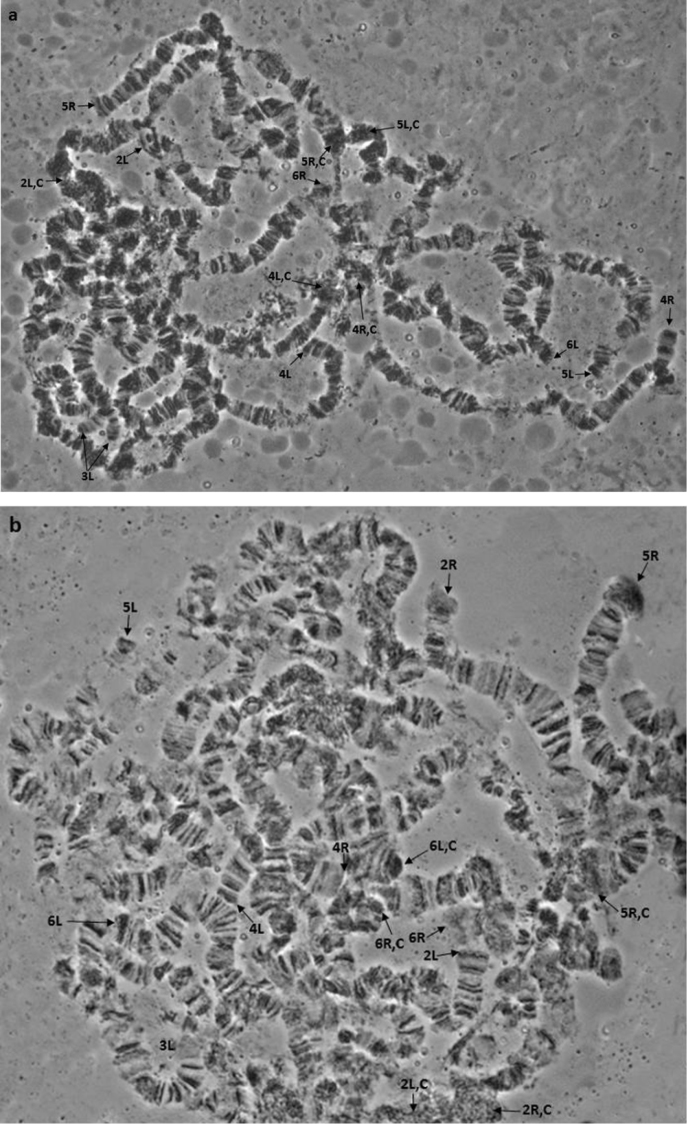
**a, b** Polytene nuclei of *Bactrocera dorsalis* *s.s.* from China. Chromosome arms are shown. Tips are marked with arrows and centromeres are indicated with ‘C’.

**Figure 2.**
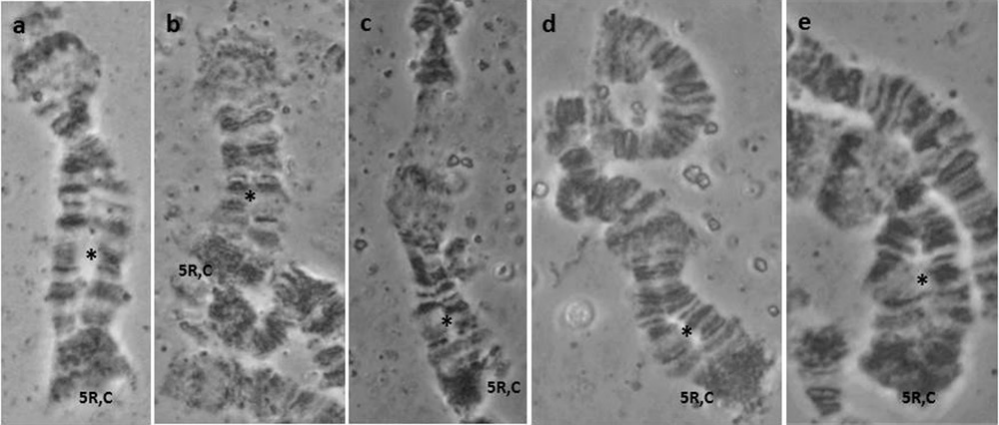
**a–e** Characteristic asynapsis in 5R chromosome arm, close to the centromere (regions 73–74), observed in the *Bactrocera dorsalis* *s.s.* colony derived from China. Asterisks (*) mark the asynaptic region, while ‘C’ marks the 5R centromere.

Polytene chromosome nuclei of *Bactrocera dorsalis* *s.s.* from China are shown in Figure 1. Its polytene chromosomes show the same banding pattern with the published maps of *Bactrocera dorsalis* *s.s.* (Zacharopoulou et al. 2011a), and therefore can be regarded as homosequential with all other colonies analysed so far (Augustinos et al. 2014b). The characteristic asynapsis at regions 73–74 of arm 5R previously observed in all colonies (Zacharopoulou et al. 2011a, Augustinos et al. 2014b) was also found here at a relatively high frequency (Figure 2). Its polymorphic presence in all colonies analysed so far points to the close genetic proximity of these five taxa.

**Figure 3.**
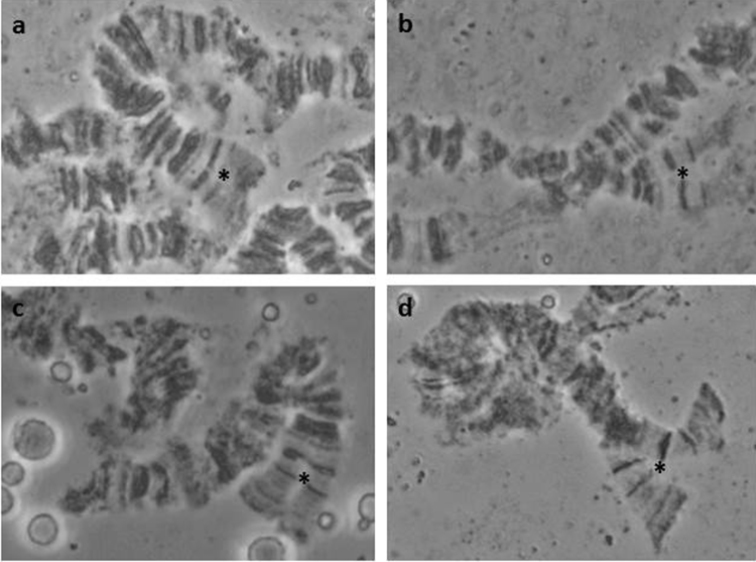
Characteristic asynapsis in the 3L, close to the tip region, observed in *Bactrocera dorsalis* colony derived from China. **a** almost completely synapsed region **b–d** asynapses of the same region; asterisks (*) indicate the specific region.

Another interesting finding from the analysis of the China colony is the high presence of an asynapsis at the telomeric region of 3L (Figure 3). Although previously observed in other colonies (Zacharopoulou et al. 2011a, Augustinos et al. 2014b), its frequency in the specific colony is much higher than in the colonies analysed before. Again, this can be considered as an inter species, intra population variation.

In the recent proposed revisions that synonymize four out of the five *Dorsalis* taxa under study (Drew and Romig 2013, Schutze et al. 2015), *Bactrocera carambolae* is maintained as a distinct species within the complex, but closely related to *Bactrocera dorsalis* *s.s.* The recent cytogenetic analysis on these five taxa failed to find any fixed diagnostic CRs among *Bactrocera dorsalis* *s.s.* and *Bactrocera carambolae* (Augustinos et al. 2014b). However, as discussed in that paper, the high frequency of small asynapses observed in the *Bactrocera dorsalis* *s.s.* × *Bactrocera carambolae* F_1_ hybrids, in comparison to the *Bactrocera dorsalis* *s.s.* × *Bactrocera invadens* F_1_ hybrids could be an indication of the presence of small CRs between the *Bactrocera dorsalis* *s.s.* and *Bactrocera carambolae* genomes, undetected with microscopy.

**Figure 4.**
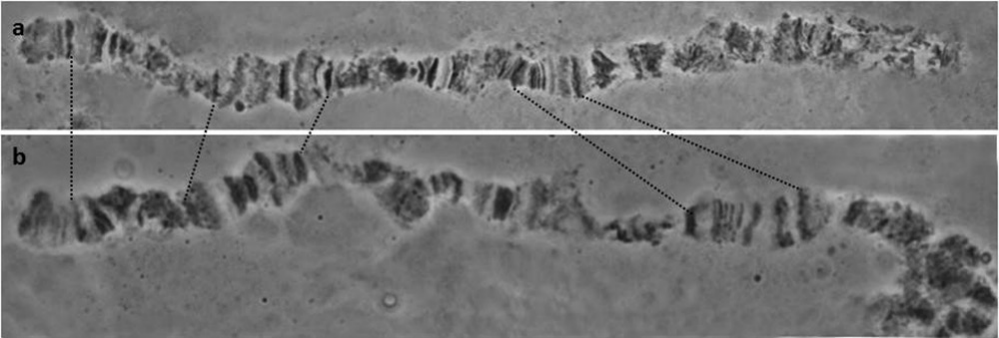
Comparison of the 3L polytene chromosome arm of **a** *Bactrocera tryoni* and **b** *Bactrocera dorsalis s.s..* Dot lines connect characteristic landmarks of the two chromosomes.

**Figure 5.**
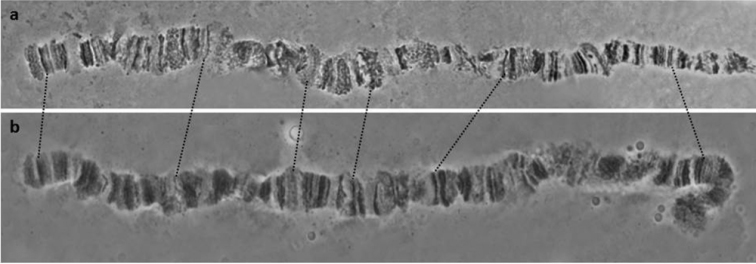
Comparison of the 4L polytene chromosome arms of **a** *Bactrocera tryoni* and **b** *Bactrocera dorsalis s.s..* Dot lines connect characteristic landmarks of the two chromosomes.

**Figure 6.**
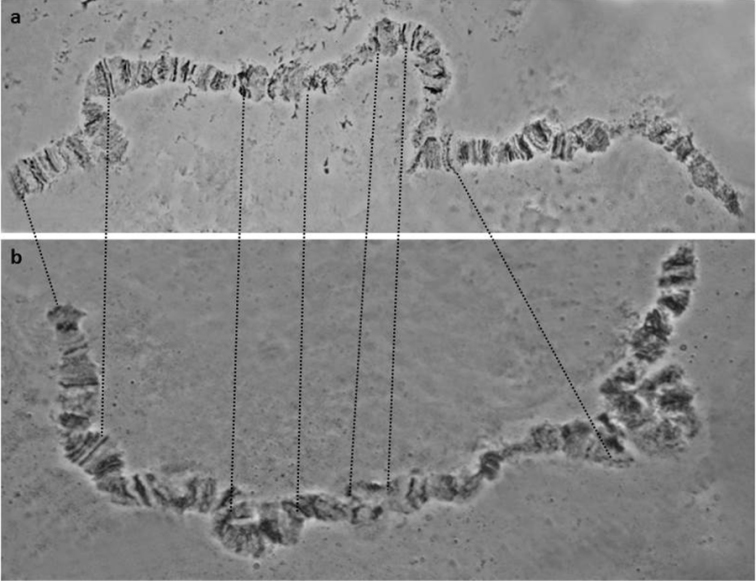
Comparison of the 5L polytene chromosome arms of **a** *Bactrocera tryoni* and **b** *Bactrocera dorsalis s.s..* Dot lines connect characteristic landmarks of the two chromosomes.

**Figure 7.**
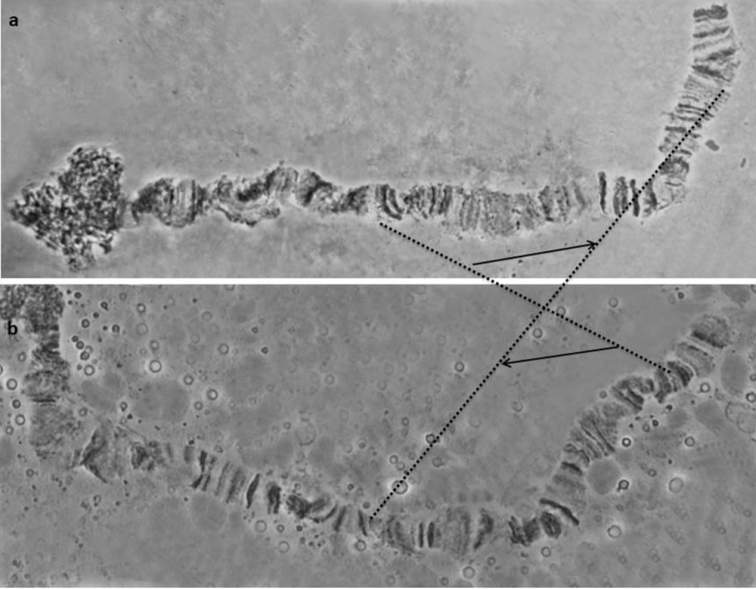
The inverted region on the 2R polytene arm that differentiates *Bactrocera tryoni* from the five members of the *Bactrocera dorsalis* complex: **a** *Bactrocera tryoni* **b** *Bactrocera dorsalis* *s.s.* Dotted lines mark the chromosomal region involved in the inversion while arrows indicate the orientation.

To explore the limitations of cytogenetic analysis in species resolution, we performed a polytene chromosome comparison between the *dorsalis* complex and *Bactrocera tryoni*, a species also belonging to the subgenus *Bactrocera* and routinely used as a closely related outgroup in different studies (Krosch et al. 2012; Boykin et al. 2014; Virgilio et al. 2015). To do so, polytene chromosome squashes from an IPCL laboratory colony were prepared and directly compared with the published *Bactrocera dorsalis* *s.s.* map (Zacharopoulou et al. 2011a), the already published *Bactrocera tryoni* map (Zhao et al. 1998) and photos from polytene chromosomes of the five taxa of the *dorsalis* complex. This analysis showed that this colony is homosequential with the previously published map of *Bactrocera tryoni*. A comparison between *Bactrocera tryoni* and the five *dorsalis* taxa showed that nine of the ten polytene arms can be regarded as highly homosequential, verifying the genetic proximity between them (Figures 4–6). However, a fixed chromosomal inversion that was previously described (Augustinos et al. 2014b), based on a comparison of polytene chromosome maps of the two species (*Bactrocera dorsalis* *s.s.* and *Bactrocera tryoni*), was verified in the new polytene chromosome squashes of the IPCL colony (Figure 7). This CR is quite extended, covering a large region of arm 2R.

**Figure 8.**
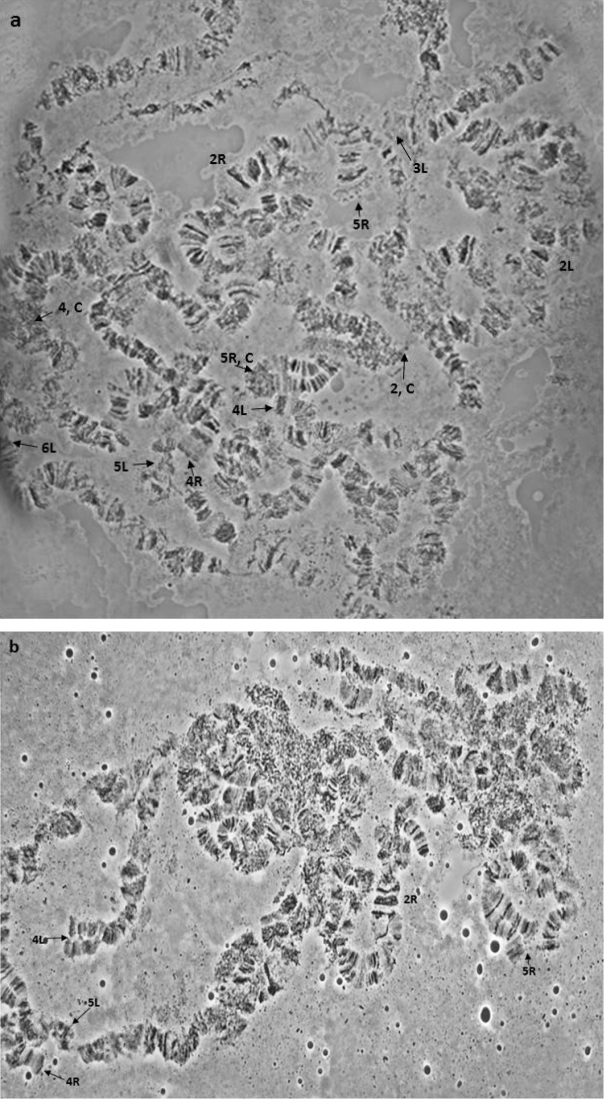
**a, b** Polytene nuclei derived from the F_1_ *Bactrocera dorsalis* *s.s.* × *Bactrocera tryoni* hybrids. Chromosome arms are indicated. Tips are marked with arrows and centromeres are indicated with ‘C’. Note the overall banding pattern homosequentiallity and the presence of limited asynapses.

**Figure 9.**
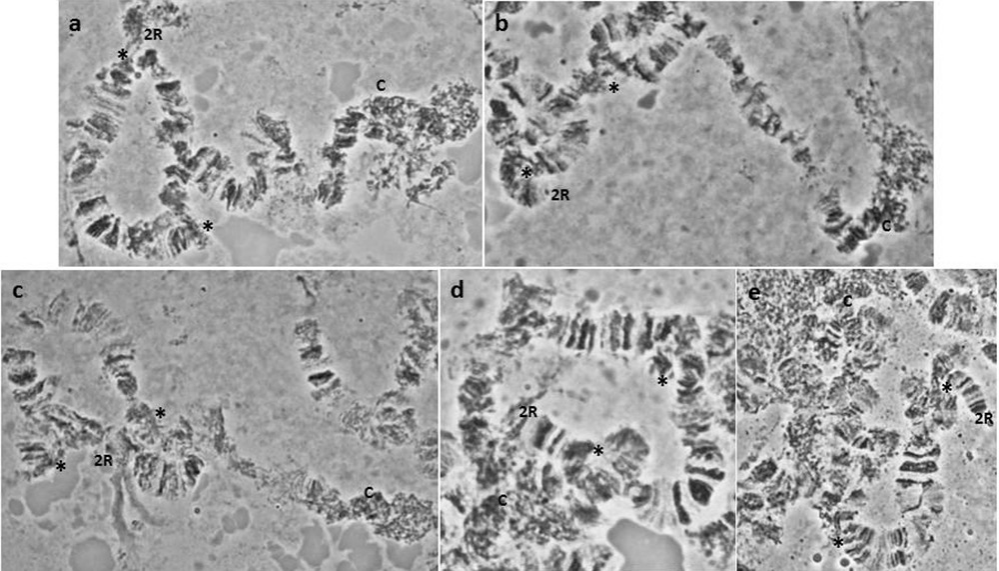
**a–e** Part of the 2R chromosome arm including the fixed inversion. Photos derived from different polytene chromosome preparations. Asterisks (*) indicate the inversion breakpoints. ‘C’ indicates the 2R centromere.

To further verify the proposed syntenies, a cytogenetic analysis of F_1_ bidirectional hybrids of *Bactrocera dorsalis* *s.s.* and *Bactrocera tryoni* was performed. Consistently with the aforementioned conclusions good synapsis can be seen in 9/10 polytene arms, while asynaptic regions are also present, as expected for hybrids of well-differentiated species (Figure 8). The inversion covering a large part of the 2R chromosome arm (Figure 8b) can also be observed, although its extension usually leads to chromosome breaks that make the mapping of breakpoints rather difficult (Figure 9).

As discussed in the Introduction, CRs are regarded as key players in Diptera speciation. In Tephritidae, all species analysed so far are differentiated by CRs, mainly inversions and transpositions. Focusing on the better studied Tephritidae species (*Ceratitis capitata*) and species of two genera that are phylogenetically close to each other (*Bactrocera* and *Dacus*), polytene chromosome comparisons performed either in older studies or in the present study have revealed specific CRs that are diagnostic in genus, subgenus and species level. Comparative analysis of the published polytene chromosome maps shows that the pericentric inversion in chromosome 5, firstly described by Zhao et al. (1998), also differentiates *Ceratitis capitata* from the other four *Dacus*/*Bactrocera* species studied so far (Zacharopoulou 1990, Mavragani-Tsipidou et al. 1992, Zacharopoulou et al. 2011a, 2011b, Drosopoulou et al. 2011b). Within the *Dacus*/*Bactrocera* clade, polytene chromosomes provide evidence for the genetic proximity of *BactroceraZeugodacus* and *BactroceraDaculus* (to a lesser extend) with *Dacus*. More specifically, there are certain CRs shared between *Bactrocera cucurbitae* (*Zeugodacus*), *Bactrocera oleae* (*Daculus*) and *Dacus ciliatus* in contrast to the two species of the *Bactrocera* subgenus (*Bactrocera dorsalis* *s.s.* and *Bactrocera tryoni*). A characteristic example is a pericentric inversion in chromosome 6 that changes the length ratio of the two arms, clearly evident when comparing the maps of these species (Mavragani-Tsipidou et al. 1992, Zhao et al. 1998, Zacharopoulou et al. 2011a, 2011b, Drosopoulou et al. 2011b). On the other hand, *Bactrocera oleae* shares also some characteristic CRs with the typical *Bactrocera* (Mavragani-Tsipidou et al. 1992, Zhao et al. 1998, Zacharopoulou et al. 2011a, 2011b, Drosopoulou et al. 2011b). Informative is also chromosome 2, since its right arm is considered as highly polymorphic among the different Tephritidae species. The region involved in the 2R inversion described before does not only differentiate *Bactrocera tryoni* from the *Bactrocera dorsalis* taxa analysed so far. This region has a unique banding pattern and/or position among the five *Bactrocera/Dacus* species analysed so far (Mavragani-Tsipidou et al. 1992, Zhao et al. 1998, Zacharopoulou et al. 2011a, 2011b, Drosopoulou et al. 2011b). All the above findings are in accordance with recent studies discussing either the genetic proximity of specific *Bactrocera* subgenera with *Dacus* or the actual status of specific subgenera, especially the *Zeugodacus* subgenus (Virgilio et al. 2009, Krosch et al. 2012, Virgilio et al. 2015).

Taking together that (a) all different Tephritidae species analysed so far exhibit characteristic CRs and (b) no diagnostic CRs could be observed in the five taxa of the *Bactrocera dorsalis* complex analysed here, it is clear that polytene chromosome analysis does so far not support a CR-mediated speciation event in the taxa under study.

### Reproductive symbiont screening – lack of evidence for symbiotic involvement in speciation events

The PCR screening for *Arsenophonus*, *Cardinium*, *Spiroplasma*, *Rickettsia* and *Wolbachia* did not reveal any signs of infection in the 19 colonies tested (Table 1). However, since this analysis was performed on populations colonized for many generations, this does not necessarily represent the ‘actual’ symbiotic status of these species in the wild. Colonization might have drastically affected the symbiotic communities of the respective populations. Although there is no evidence for the implication of reproductive symbionts on speciation events between the investigated taxa, symbiotic analysis of wild populations is thus crucial to fully resolve the symbiotic status of these taxa and the *dorsalis* complex in general.

In Tephritidae, only *Wolbachia* has so far been found in a limited number of species, while there are no reports of the presence of the other four symbionts. This can partly be attributed to a lack of comprehensive surveys. Regarding the *Bactrocera dorsalis* complex, there are reports for the presence of *Wolbachia* in natural populations (Kittayapong et al. 2000, Jamnongluk et al. 2002, Sun et al. 2007), however only a few populations and at very low frequencies. The first of them (Kittayapong et al. 2000) reports a *Wolbachia* PCR screening of fruit flies of Thailand, collected in the years 1995-1998. Screening was based on the *ftsZ* gene and supergroup-typing on *wsp* sequences. Only 2/222 of the mitotic form A samples and one out of two of the mitotic form K samples were infected. The infection was reported as belonging to supergroup B. Later on, the same research group, using the same samples, suggested the presence of multiple *Wolbachia* infections (Jamnongluk et al. 2002). More recently, a study performed on Chinese populations of *Bactrocera dorsalis* *s.s.* (Sun et al. 2007) revealed very low levels of *Wolbachia* infections (19 positive samples of 1500), belonging either to supergroup A or B, based on *wsp* sequencing. Given the available knowledge at the time of these screens, the specimens tested might have not been properly identified at the species level. In any case, it is highly unlikely that at such low frequencies *Wolbachia* infection could trigger or support a speciation event.

## Conclusion

CRs are a well-known indicator of speciation in Diptera, while symbionts obtain only during the last years more recognition as putative speciation factors. Analysing possible paths of speciation with multidisciplinary approaches (integrative taxonomy) is now acknowledged as the best way to provide robust results in species delimitation (De Queiroz 2007, Schlick-Steiner et al. 2010). Our analysis, focused on five economically important members of the *Bactrocera dorsalis* complex currently colonized at the Joint FAO/IAEA IPCL, failed to identify any fixed CRs or specific reproductive symbionts that could have participated in the speciation process in the complex. These results are in line with recent data that question the ‘actual’ number of species within the *Bactrocera dorsalis* complex (Krosch et al. 2013, Schutze et al. 2013, San Jose et al. 2013, Boykin et al. 2014) and have led to the recent synonymization proposed by Schutze and colleagues (Schutze et al. 2015). Analysis of species within the complex that are more clearly differentiated from the five taxa under study could shed more light on the speciation process within the *Bactrocera dorsalis* complex.
